# Gray matter reduction is associated with cognitive dysfunction in depressed patients comorbid with subclinical hypothyroidism

**DOI:** 10.3389/fnagi.2023.1106792

**Published:** 2023-02-08

**Authors:** Shuai Zhao, Yishan Du, Yu Zhang, Xiaoqin Wang, Yi Xia, Hao Sun, Yinghong Huang, Haowen Zou, Xumiao Wang, Zhilu Chen, Hongliang Zhou, Rui Yan, Hao Tang, Qing Lu, Zhijian Yao

**Affiliations:** ^1^Department of Psychiatry, The Affiliated Psychological Hospital of Anhui Medical University, Hefei, China; ^2^Hefei Fourth People’s Hospital, Hefei, China; ^3^Anhui Mental Health Center, Hefei, China; ^4^Anhui Clinical Research Center for Mental Disorders, Hefei, China; ^5^Department of Psychiatry, The Affiliated Brain Hospital of Nanjing Medical University, Nanjing, China; ^6^Nanjing Brain Hospital, Medical School of Nanjing University, Nanjing, China; ^7^School of Biological Sciences & Medical Engineering, Southeast University, Nanjing, China; ^8^Child Development and Learning Science, Key Laboratory of Ministry of Education, Nanjing, China

**Keywords:** gray matter, cognitive decline, MDD, subclinical hypothyroidism, fMRI

## Abstract

**Introduction:**

To explore the association between regional gray matter volume (GMV) and cognitive impairments and ascertain whether the regional brain alterations related to cognitive impairments occur in major depressive disorder (MDD) patients with comorbid subclinical hypothyroidism (SHypo).

**Methods:**

We enrolled 32 MDD patients, 32 MDD patients with comorbid SHypo, and 32 normal controls and subjected them to thyroid function tests, neurocognitive tests, and magnetic resonance imaging (MRI). Using voxel-based morphometry (VBM) analysis, we examined the pattern of gray matter (GM) in these participants. We also used ANOVA to detect group differences and partial correlation to explore the potential association between GMV alterations and cognitive tests in comorbid patients.

**Results:**

The comorbid patients exhibited significantly smaller GMV in the right middle frontal gyrus (MFG) than the non-comorbid group. Furthermore, the partial correlation analysis showed that GMV of the right MFG was associated with poor executive function (EF) performance in comorbid patients.

**Conclusion:**

These findings provide valuable insight into the relationship between the alteration of GMV and cognitive dysfunction of MDD patients with comorbid SHypo.

## 1. Introduction

Major depressive disorder (MDD) is a common psychotic disorder characterized by lower mood, sluggish thinking, and reduced tolerance for physical activity. Epidemiological research suggests that depression constitutes the leading cause of disability worldwide (Collaborators, 2022). With people’s living standards improving, MDD has become a significant threat to human health, with a high incidence rate, recurrence rate, disability rate, and an enormous economic burden. Although some achievements have been made in the acute-phase treatment of MDD, there are still some deficiencies in long-term treatment. Examples are insufficient drug dose and treatment course, frequent and unnecessary adjustment of drug treatment, and poor adherence to treatment (Herrman et al., 2022). Most antidepressants used clinically act mainly through neurotransmitters (i.e., monoamine, serotonin, and noradrenaline; Berton and Nestler, 2006). However, many studies have shown that the pathogenesis of depression also involves neuroendocrine disturbances and immune-inflammatory and metabolic abnormalities (Horowitz and Zunszain, 2015). Of these, the hypothalamic–pituitary-thyroid (HPT) axis was thought to take part in the pathophysiology of depression (Nemeroff and Evans, 1989). On the one hand, the HPT axis dysfunction can cause emotional and psychotic symptoms, possibly due to thyroid hormone interference with metabolic processes and intracellular signaling pathways (Glombik et al., 2021). On the other hand, abnormal thyroid hormone levels are known to negatively affect cognitive function (Uter et al., 2020).

Cognitive deficits are a core feature of MDD, associated with depression and related to many psychosocial issues, negatively affecting academic achievement, social functioning, and mental development (Kupferberg et al., 2016; Suciu and Miclutia, 2020). Despite cumulating evidence regarding persistent cognitive impairment in MDD, research on treatment options specific to this patient group is still scarce (Listunova et al., 2018). Interestingly, there has been some evidence that thyroid disease can alter cognitive function, although the exact mechanism remains elusive (Bauer et al., 2008; Ceresini et al., 2009).

Subclinical hypothyroidism (SHypo) is identified by elevated serum thyroid-stimulating hormone (TSH) levels and normal concentrations of free thyroxine (FT4; Cooper and Biondi, 2012). The relationship between SHypo and MDD is largely unclear (Karakatsoulis et al., 2021). An extensive study (*n* = 92,206) revealed no association between the presence of SHypo and the development of depression during a mean follow-up of 2 years (Kim et al., 2018). Another relatively recent study also found no association between SHypo and MDD in adolescents (Hirtz et al., 2022). In contrast, in another two recent meta-analyses, including case–control studies assessing more severe cases from clinical settings, 12,315 (Loh et al., 2019) and 103,375 (Tang et al., 2019) adults were found to have a higher risk of SHypo in MDD. Heterogeneous study populations, small sample size, lack of a control arm comparison, and different study designs may explain the inconsistent results. Besides, SHypo has been reported to be associated with cognitive impairments, including attention, memory, and executive functions (Zhu et al., 2006; Pasqualetti et al., 2015). A functional magnetic resonance imaging (fMRI) study showed that SHypo patients with impaired working memory exhibited less activation of the middle frontal gyrus (MFG) and supplementary motor area (SMA) during the n-back task than healthy controls (Zhu et al., 2006). Another study found that SHypo had significantly longer reaction times and lower performance accuracy when using the Stroop test, suggesting that they may have impaired attentional control function (Yin et al., 2021). The possible mechanism might be related to the glial cell-mediated action of thyroid hormones (THs) on the brain, affecting neuronal proliferation, migration, and differentiation (Tost et al., 2020). Notably, several studies have shown that patients with MDD have a high prevalence of SHypo (Lang et al., 2020). Our previous study also reported similar results (Zhao et al., 2021a). Whether the comorbidity of the two diseases may worsen patients’ cognitive impairment deserves the attention of researchers and clinicians.

Recently, with the development and broad adoption of neuroimaging, we can understand the comorbidity of the two diseases in light of brain mechanisms. For instance, overall gray matter volume (GMV) reductions in MDD and SHypo patients were found separately in previous studies. One study indicated that a subclinical hypothyroidism state might reduce brain volume, particularly the hippocampal (Ittermann et al., 2018). A study using voxel-based morphometry (VBM) found that patients with SHypo had less GMV in the bilateral prefrontal gyrus (Yin et al., 2021). Another follow-up study demonstrated that the GMV reduced in patients with SHypo tended to improve after levothyroxine replacement therapy (Zhang et al., 2021). For MDD patients, most studies have also identified decreased GMV in a wide range of brain regions (Peng et al., 2011; Zhang et al., 2012; Lai and Wu, 2014; Zheng et al., 2021). Therefore, whether GMV was reduced in MDD patients comorbid with SHypo is unclear. Moreover, numerous studies confirmed that brain structural alterations could affect cognitive functions in healthy and patient populations (Zimmerman et al., 2006; Dalaker et al., 2010). Whether the decreased GMV might negatively influence comorbid patients’ cognitive performance remains elusive.

Accordingly, this study focused on MDD patients comorbid with SHypo, exploring cognitive performance and the potential role of GMV alterations. Our first goal was to assess cognitive performance using a comprehensive neuropsychological battery of tests in all participants. The second goal was to examine GMV differences among MDD patients, comorbid patients, and healthy controls. The third goal was to explore the potential association between GMV alterations and cognitive tests in comorbid patients. We speculate that MDD patients comorbid with SHypo may demonstrate decreased GMV, negatively influencing their cognitive performance.

## 2. Methods

### 2.1. Participants

Our sample comprised three groups of participants, i.e., 32 MDD patients comorbid with SHypo (comorbid group), 32 MDD patients (non-comorbid group), and 32 healthy controls (HCs group). From July 2019 to February 2021, all patients with MDD were recruited from the Department of Psychiatry of the Affiliated Nanjing Brain Hospital of Nanjing Medical University. The following were the inclusion criteria for this population: (1) Diagnosis of MDD was established according to the Diagnostic and Statistical Manual of Mental Disorders, 5th edition (DSM-V) criteria, either single episode or recurrent episodes, (2) between the ages of 18 and 55, (3) right-handed, and (4) education level ≥8 years. Two experienced psychiatrists interviewed all consenting subjects. MDD severity was assessed using the 17-item Hamilton Scale for Depression (HAMD; Hamilton, 1960). All patients were drug-free for at least 2 weeks before the MRI. Study patients with any of the following conditions will be excluded: (1) psychopathologies other than MDD, (2) neurologic or organic brain disease, (3) substance abuse/dependence within the previous year, (4) current pregnancy or breastfeeding, (5) history of severe physical illness or contraindication to MRI. Meanwhile, 32 HCs were recruited *via* advertisement. All participants were administered the Mini International Neuropsychiatric Interview (MINI; Sheehan et al., 1998) to confirm the diagnosis of MDD and to rule out current or past psychiatric illness in healthy controls.

After providing subjects with a complete description of the study, written informed consent was obtained. The Research Ethics Review Board approved the study of the Affiliated Nanjing Brain Hospital of Nanjing Medical University.

### 2.2. Clinical and neurocognitive assessment

Participants completed a self-report questionnaire describing their socio-demographic characteristics, including age, gender, education, and marital status. The first episode or recurrence, as well as mental illness, were considered clinical variables. HAMD measures the severity and frequency of depression symptoms in individuals based on a 17-item scale. Scores of 0–7 are considered normal, while scores of 18 or more indicate moderate severity. Anxiety was assessed using the 14-item Hamilton Anxiety Rating Scale (HAMA).

A comprehensive battery of neuropsychological tests was administered to all participants. We divided the subtests of these tests into four major domains based on the previous literature: attention, memory, processing speed, and executive function (Liu et al., 2019). A Trail Making Test (TMT)-A was conducted to assess attention (Reitan, 1958). Memory function was divided into verbal and visual memory separately. We used the Wechsler Memory Scale-Revised (WMS-R) subsets of logical and figural memory (Wechsler, 1987) to assess verbal and visual memory. The Digital Symbol Substitution Test (DSST) evaluated processing speed (Wechsler, 1955). We measured executive function using two different tests. Digit Span Backward (DSB) measures working memory, and TMT-B assesses cognitive flexibility (Wechsler, 1997). These tests were chosen due to their frequent use in previous studies on MDD in adults and adolescents (Russo et al., 2015; Liu et al., 2019; Zhao et al., 2021b).

### 2.3. Serum THs levels assessments

Subjects were fasted overnight (8 h) before collecting peripheral blood samples. TSH, free triiodothyronine (FT3), and free thyroxine (FT4) were measured using the electrochemical luminescence method (Roche Company Cobas E601 automatic immunoassay). SHypo was defined as TSH > 4.2 mIU/L with FT4 levels within the reference ranges. The reference interval of TSH was 0.27–4.2 mIU/L. The reference interval of FT4 values was 12–22 pmol/L (Rogowicz-Frontczak et al., 2020; Zhao et al., 2021a).

### 2.4. MRI data acquisition and preprocessing

A high-resolution T1-weighted structural image was performed using a three-dimensional gradient-echo sequence for anatomical reference. We provided soft earplugs and positioned the participants comfortably on the scanner table. Foam pads stabilized their heads and minimized movement. The imaging parameters for the T1 mapping MRI was set as follows: repetition time (TR) = 1,900 ms, echo time (TE) = 2.48 ms, flip angle (FA) = 9°, 176 axial slices with thickness = 1 mm, gap = 0 mm, in-plane voxel resolution = 1 mm × 1 mm, and field of view (FOV) = 25 × 25 cm^3^. Artifacts and structural abnormalities were examined in all images.

### 2.5. Voxel-based morphometry analysis

All the T1-weighted brain structural images were processed using CAT12^1^ based on the SPM12 software,^2^ a widely used program for performing VBM analyses. The default settings were used unless otherwise specified. First, quality control of image registrations was performed by visual inspection. Second, the origin of an image should be set to the anterior commissure for better image registration. Third, the original individual T1-weighted images were segmented into gray matter (GM), white matter (WM), and cerebrospinal fluid (CSF) using a unified segmentation approach (Ashburner and Friston, 2005). Fourth, GM and WM segmented images from all subjects to create a study-specific template with Diffeomorphic Anatomical Registration Through Exponentiated Lie (DARTEL). Next, using modulation, segmented images were warped to study-specific templates and spatially normalized to Montreal Neurological Institute (MNI) space. Lastly, we smoothed the modulated gray matter images with an 8 mm full width at half maximum (FWHM) isotropic Gaussian kernel. The total intracranial volume (TIV) was calculated by summing the total GM, WM, and CSF volumes. Normalized volumes for gray matter (nGMV) were calculated by dividing by the TIV and expressed as percentages.

### 2.6. Statistical analysis

The data were analyzed using SPSS 19.0 software. Continuous variables were presented as means ± SD, and categorical variables were displayed as percentages. We used a one-way variance analysis (ANOVA) to compare the demographic, THs, nGMV, TIV, and cognitive performance. Least significant differences (LSD) *post-hoc*-tests evaluated *post-hoc* comparisons. A two-sample *t*-test was used to compare HAMD and HAMA between the comorbid and non-comorbid groups. Two-tailed significant levels were set at 0.05.

We used a one-way analysis of covariance (ANCOVA) to identify GM density changes among the three groups. In order to identify differences between each pair of groups, *post-hoc t*-tests were conducted. Age, gender, education, and TIV were covariates of no interest. All analyses were performed using cluster inference with a cluster-defining *p* < 0.001 and a cluster-probability threshold of *p* < 0.05 family-wise error (FWE) corrected for multiple comparisons. To investigate the relationship between THs, GMV, and cognitive tests in the comorbid patient group, the GMV values that differed significantly between the two depression groups were extracted and related to THs, and cognitive tests (group differences in the two depression groups). Years of education, age, and gender were used as the covariates. A partial correlation analysis was not corrected for multiple testing to investigate potential associations.

## 3. Results

### 3.1. Demographic information

The demographics and clinical characteristics of all participants are given in Table 1. This study enrolled 64 MDD patients, including 32 in the comorbid and 32 in the non-comorbid groups. There were no significant differences between the groups regarding age, sex, years of education, and marriage (*p* > 0.05). Moreover, no differences in the recurrence, HAMD, and HAMA scores between the two MDD groups were found (*p* > 0.05). Comorbid patients had TSH levels higher than the upper limit of the normal range, and the difference among them was significant (*F* = 105.595, *p* < 0.001). There was no significant difference between the three groups regarding the FT3 and FT4 levels (*p* > 0.05).

**Table 1:** The demographics, clinical characteristics, and cognitive function of the three groups of participants.

Items	Non-comorbid	Comorbid	HC	*F*/*χ*^2^/*t*	*p*
	*n* = 32	*n* = 32	*n* = 32		
Age, years	28.6 ± 12.6	33.4 ± 11.7	31.3 ± 8.3	1.537	0.220
Gender, male/female	15/17	12/20	15/17	0.762	0.683
Education, years	13.1 ± 2.9	13.8 ± 3.9	14.3 ± 2.0	2.373	0.099
Married, *n* (%)	37.5%	62.5%	53.1%	4.085	0.130
Recurrence, *n* (%)	59.4%	71.9%	NA	1.108	0.292
HAMD scores	25.6 ± 6.7	24.2 ± 6.3	NA	0.866	0.390
HAMA scores	13.7 ± 6.3	16.7 ± 7.9	NA	−1.647	0.105
TSH (mIU/L)	1.8 ± 0.9	6.0 ± 1.8	2.3 ± 0.8	105.595	<0.001*
FT3 (pmol/L)	4.7 ± 0.8	4.8 ± 0.9	4.7 ± 0.6	0.102	0.903
FT4 (pmol/L)	16.3 ± 3.1	17.2 ± 10.5	16.5 ± 1.82	0.204	0.816
nGMV (ml)	0.44 ± 0.03	0.43 ± 0.03	0.45 ± 0.02	4.307	0.016*
TIV (ml)	1469.2 ± 116.7	1391.3 ± 119.7	1501.7 ± 99.8	8.138	0.001*
Attention					
TMT-A	38.3 ± 11.6	46.5 ± 17.4	29.8 ± 8.4	13.137	<0.001*
Processing speed					
DSST	50.9 ± 10.1	52.3 ± 11.9	58.9 ± 5.3	6.408	0.002*
Verbal memory					
WMS-LM	10.1 ± 2.8	11.1 ± 3.4	13.1 ± 3.1	7.805	0.001*
Visual memory					
WMS-FM	14.6 ± 2.8	14.3 ± 3.2	16.4 ± 2.8	5.059	0.008*
Cognitive flexibility					
TMT-B	56.0 ± 12.5	61.2 ± 30.0	38.7 ± 12.6	10.614	<0.001*
Working memory					
DSB	6.6 ± 1.7	5.6 ± 1.8	7.4 ± 1.5	9.538	<0.001*

HC, Healthy Control; HAMD, Hamilton Rating Scale for Depression; HAMA, Hamilton Rating Scale for Anxiety; FT3, Free Triiodothyronine; FT4, Free Thyroxine; TSH, Thyroid-stimulating Hormone; nGMV, normalized Volumes for Gray Matter; TIV, Total Intracranial Volume; TMT-A, Trail Making Test A; DSST, Digital Symbol Substitution Test; WMS-LM, logical memory subset of the Wechsler Memory Scale; WMS-FM, logical memory subset of the Wechsler Memory Scale; TMT-B, Trail Making Test B; DSB, Digit Span Backward; Comparisons were conducted using *t*-tests (*t*), ANOVA tests (*F*), and chi-squared tests (*χ*^2^).

* *p* < 0.05.

### 3.2. Cognitive function results

As shown in Table 1, there were significant differences in cognitive function scores among the three groups (*p* < 0.05). *Post-hoc* analysis further identified that the comorbid group showed significantly worse performance in attention (*p* = 0.013) and EF (*p* = 0.018) than the non-comorbid group.

### 3.3. VBM results

**Figure 1:**
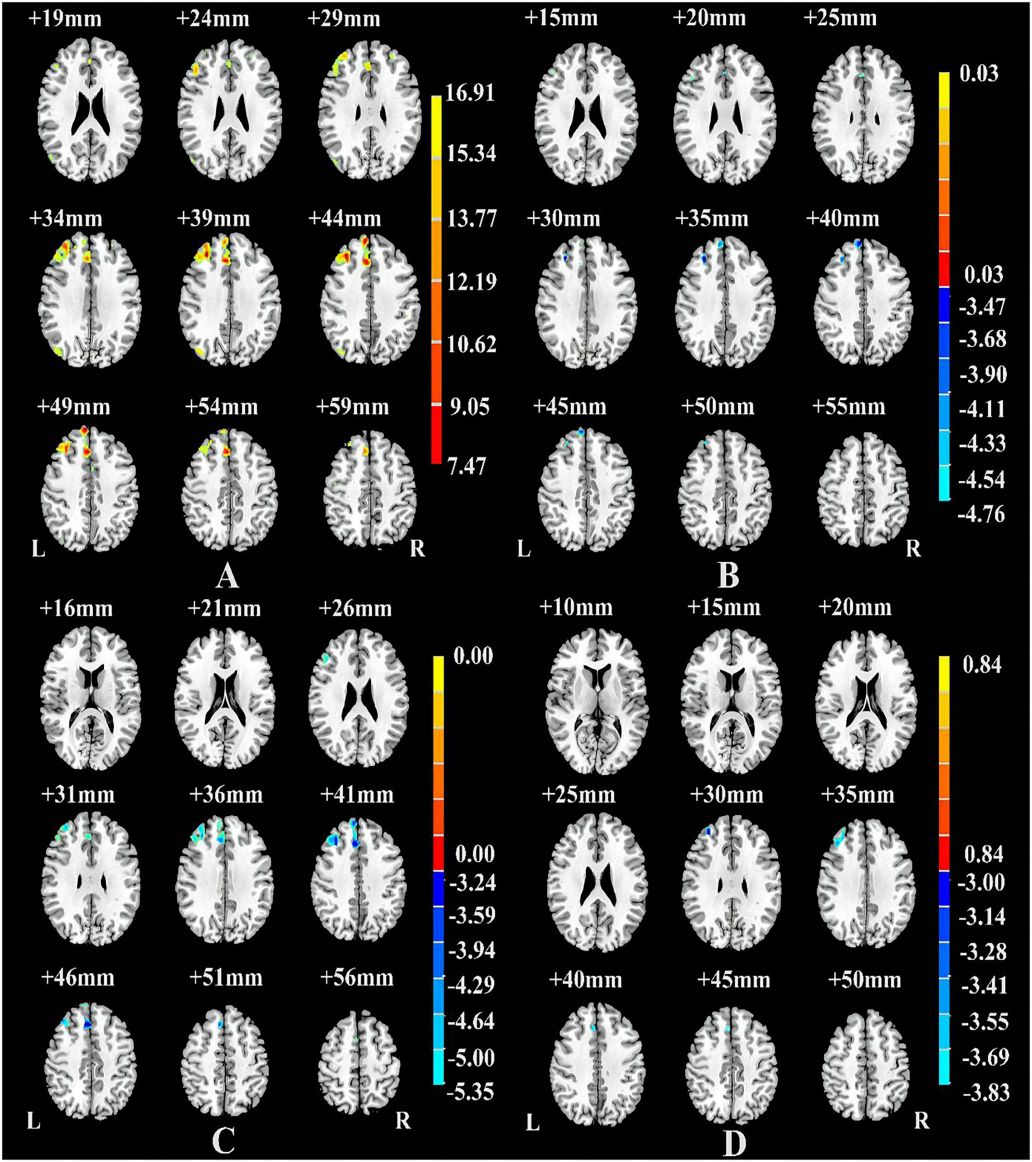
Brain regions showing differences among the Comorbid, non-Comorbid, and HC groups **(A)**, between the non-Comorbid group and HC groups **(B)**, between the Comorbid group and HC groups **(C)**, between the non-Comorbid group and Comorbid groups **(D)**. *p* < 0.05, corrected for multiple comparisons using FWE cluster-wise corrected.

Table 1 shows significant differences in nGMVs among the three groups (*p* < 0.05). *Post-hoc* analysis further identified that the comorbid group showed significantly smaller GMV (*p* = 0.013) and TIV (*p* = 0.007) than the non-comorbid group. As shown in Figure 1A and Table 2, ANCOVA analysis revealed three significantly different clusters among the three groups: right medial superior frontal gyrus (mSFG), right MFG, and right SMA (*p* < 0.05, FWE cluster-wise corrected).

**Table 2:** Brain areas with significantly different GMV among the comorbid, non-comorbid, and HC groups.

Peak location	Cluster size (voxels)	Peak MNI coordinates			*F*/*t* values
		*x*	*y*	*z*	
Three groups					
Frontal_Sup_Medial_R	1,101	6	52.5	42	15.7194^a^
Frontal_Mid_R	1,338	31.5	34.5	37.5	16.9133^a^
Supp_Motor_Area_R	861	6	10.5	58.5	13.3471^a^
Non-comorbid vs. HCs					
Frontal_Mid_R	128	28.5	34.5	36	−4.7591^b^
Frontal_Sup_Medial_R	150	6	52.5	42	−4.5635^b^
Comorbid vs. HCs					
Frontal_Mid_R	1,158	31.5	34.5	37.5	−5.0193^b^
Supp_Motor_Area_R	532	3	9	69	−4.0693^b^
Comorbid vs. non-comorbid					
Frontal_Mid_R	80	31.5	45	28.5	−3.832^b^

*x*, *y*, *z* are the coordinates of primary peak locations in the MNI space; *F* is obtained by one-way analysis of covariance; *t* is obtained by two-sample two-tailed *t*-test (FWE cluster-wise corrected, *p* < 0.05); L, left, R, right.

a The *F* statistical value.

b The *t* statistical value.

Compared with the HC group, the non-comorbid group exhibited significantly smaller GMV in the right mSFG and MFG (Table 2; Figure 1B); the comorbid group exhibited significantly smaller GMV in the right MFG and mSFG (Table 2; Figure 1C). Compared with the non-comorbid group, the comorbid group showed significantly smaller GMV in the right MFG (Table 2; Figure 1D; *p* < 0.05, FWE cluster-wise corrected).

### 3.4. Exploratory correlational analyses among THs, GMVs, and cognitive tests in the comorbid group

**Figure 2:**
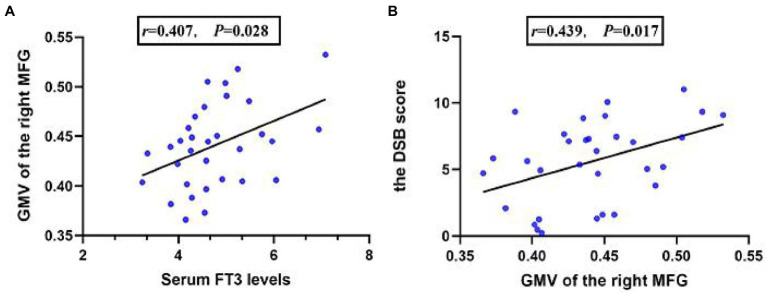
The partial correlation between serum FT3 levels and the GMV of the right MFG in comorbid patients **(A)**, the partial correlation between the GMV of the right MFG, and the DSB score in comorbid patients **(B)**.

In comorbid patients, serum FT3 levels were positively correlated with GMV of the right MFG (*r* = 0.422, *p* = 0.023; Figure 2A). In addition, the GMV of the right MFG was positively correlated with the DSB score in comorbid patients (*r* = 0.439, *p* = 0.017; Figure 2B). No association was found in the non-comorbid and HC group (*p* > 0.05).

## 4. Discussion

The involvement of THs in the pathogenesis of depression is indicated by some symptoms observed in depression and SHypo. Cognitive impairment is also a characteristic and frequently observed manifestation in both diseases. Given this, we examined the cognitive performance in MDD patients comorbid with SHypo and its underlying brain mechanisms. The current study revealed three major findings. Firstly, attention and EF deficits were more severe in comorbid patients than in non-comorbid patients. Secondly, comorbid patients showed significantly smaller GMV in the right MFG than non-comorbid patients. Thirdly, partial correlation analysis showed that serum FT3 levels were related to GMV of the right MFG, which was associated with poor EF performance.

Recently, several observational studies have reported that MDD patients comorbid with SHypo presented higher Body Mass Index (BMI; Kang et al., 2022), more severe anxiety, and increased suicide attempts (Shangguan et al., 2022). Our findings may contribute to better comprehending the clinical features of MDD patients comorbid with SHypo. We found that the cognitive impairment of comorbid patients is mainly reflected in attention and EF, which may be related to SHypo. The cognitive impairment associated with SHypo is usually part of a broader psychopathological picture, which includes concentration disorders, mood changes, and EF. A recent fMRI study using the Stroop test showed that patients with SHypo had a significantly longer reaction and lower performance accuracy, indicating impaired attentional function (Yin et al., 2021). Another recent study found that patients with thyroid dysfunction exhibited damaged altering networks revealed by the attention network test (ANT; Yuan et al., 2020). In addition, Zhu et al. (2006) found reduced bilateral dorsolateral prefrontal cortex (DLPFC) activation with fMRI during *n*-back tasks, suggesting that patients with SHypo exhibited impaired EF. More importantly, deficits in attention and EF may impair the ability to shift attention and cope with strategies when confronted with negative emotions (Liu et al., 2019), which may aggravate their symptoms.

It is worth noting that TSH plays a crucial role in regulating neuronal differentiation and synaptic plasticity (Horn and Heuer, 2010). Low thyroid hormone levels in the pituitary and high TSH levels would likely result in even lower thyroid hormone levels in the brain (Cohen et al., 2018). Thus, a high TSH level requires serious consideration. More importantly, abnormal TSH levels modulate various cerebral functions involved in cognition and mood. An earlier study found that TSH negatively impacted memory function in healthy aging adults (Wahlin et al., 1998). In another study, individuals with TSH levels within normal ranges were at higher risk for Alzheimer’s disease (van Osch et al., 2004). We speculate that cognitive impairment might be associated with high TSH levels in comorbid patients. Regrettably, no associations between TSH levels and cognitive tests were found in this study, limiting the interpretation of the results.

Thus far, no study has specifically addressed brain MRI abnormalities in MDD patients comorbid with SHypo. In our VBM study, comorbid patients showed a significantly decreased GMV than the non-comorbid group, and these brain regions were primarily located in the right MFG. It is widely recognized that THs are essential for both the development and maturation of the cerebrum and affect such diverse events as neuronal integration and processing, myelination, and glial cell proliferation. A large United Kingdom Biobank study demonstrated that thyroid status could affect adult GMV (Chambers et al., 2021). Another recent study showed that low maternal thyroid function during pregnancy is associated with minor child gray matter (Jansen et al., 2019). In this context, we hypothesize that the altered HPT of comorbid patients affects the GMVs of the MFG. More importantly, our results revealed that reduced GM density in the right MFG was associated with serum FT3 levels. Our previous study also demonstrated an inverse relationship between TSH levels and the GMV of MFG (Zhao et al., 2021c). Although the associations could not extend casual pathophysiology, changes in THs may impair the GMV of MFG, and induce cognitive dysfunction. However, future longitudinal studies are needed to address this speculation.

To our knowledge, no published studies combine cognitive functioning and MRI of the brain in MDD patients comorbid with SHypo. The present study may provide some clues regarding the causes of cognitive deficits in MDD patients comorbid with SHypo, as DSB scores were associated with the GMV of the right MFG. MFG is a critical component of the DLPFC and is involved in attention and working memory (Sanchez-Lopez et al., 2018; Jones and Graff-Radford, 2021). Impairment of the DLPFC may elicit executive dysfunction in MDD patients comorbid with SHypo. Combining the above results, we hypothesize that dysfunction in the HPT axis may contribute to GMV loss in the frontal lobe, which further impairs EF. Nevertheless, the results of this study should be interpreted cautiously.

Additionally, we did not find any difference in depressive symptoms between comorbid and non-comorbid patients. A previous study reported that MDD patients who had attempted suicide or exhibited symptoms of psychiatric illness were more likely to have SHypo (Lang et al., 2020). However, our previous cross-sectional study found no differences in symptom severity between comorbid and non-comorbid patients (Zhao et al., 2021a). This difference could be related to the different sample sizes and diagnostic processes. Likewise, no associations between HAMD scores and cognitive function tests were found in comorbid and non-comorbid patients. Following the results, cognitive function impairment is not an epiphenomenon caused solely by depression severity but rather a core feature of depression. Deficits in cognitive function may be essential trait markers of MDD, as they persist even after depressive symptoms subside (Lee et al., 2012). Although thyroid hormones have not been adequately investigated as a pro-cognitive treatment for MDD patients, several studies implied that levothyroxine seems to reverse cognitive impairment caused by thyroid dysfunction (Tost et al., 2020; Bauer and Whybrow, 2021). A recent study also showed that the corresponding structural and functional deficiencies tended to improve after thyroid hormone therapy in patients with SHypo (Zhang et al., 2021). More longitudinal studies are needed to provide a complete picture of the interrelationship between thyroid hormone therapy, gray matter alterations, and cognitive dysfunction. In addition, there was no such association in HCs, indicating that the observed relationships are unique to comorbid patients.

We acknowledge that our study has some limitations. Firstly, causality between variables cannot be determined in a cross-sectional study. Secondly, the sample size is relatively small, and a larger is required. Thirdly, not all cognitive functions are evaluated despite the comprehensive neuropsychological assessment. Fourthly, the comorbid patients were not followed up simultaneously, so it was unclear whether the GMV and cognitive function could recover to normal after treatment of SHypo. Longitudinal perspectives could help confirm the relationship between thyroid function, structure changes in the brain, and cognitive decline. Finally, residual confounding cannot be excluded despite carefully considering potential confounders.

## 5. Conclusion

The present study aimed to analyze the possible link between gray matter changes and cognitive impairment in MDD patients comorbid with SHypo. Our results indicate that MDD patients comorbid with SHypo exhibited reduced gray matter density and cognitive dysfunction. Furthermore, there is a correlation between serum FT3 levels and regional GMVs within MFG in comorbid patients. Importantly, comorbid patients who exhibited reduced GM may negatively influence EF. These findings provide valuable insight into the relationship between the alteration of GMV and the cognitive dysfunction of comorbid patients. Therefore, more attention needs to be paid to MDD patients with comorbid SHypo, and the importance of routine thyroid function examination in MDD patients cannot be underestimated.

## Data availability statement

The original contributions presented in the study are included in the article/supplementary material, further inquiries can be directed to the corresponding authors.

## Ethics statement

The studies involving human participants were reviewed and approved by the Ethics Committee of the Affiliated Brain Hospital of Nanjing Medical University. The patients/participants provided their written informed consent to participate in this study.

## Author contributions

SZ and ZY conceived of the presented idea. YD, ZC, and QL contributed to the design and implementation of the research. XMW, YX, and RY carried out experiments. HLZ, HS, HWZ, and XQW performed the analytic calculations. SZ, YZ, HT, and YH revised the manuscript. All authors contributed to the article and approved the submitted version.

## Funding

This study was supported by grants of the National Natural Science Foundation of China (81871066); the Jiangsu Provincial Key Research and Development Program (BE2018609 and BE2019675); the Jiangsu Provincial Medical Innovation Team of the Project of Invigorating Health Care through Science, Technology and Education (CXTDC2016004); the Key Project supported by Medical Science and Technology Development Foundation, Jiangsu Commission of Health (K2019011); and Anhui Provincial Medical and Health Key Specialties Project.

## Conflict of interest

The authors declare that the research was conducted in the absence of any commercial or financial relationships that could be construed as a potential conflict of interest.

## Publisher’s note

All claims expressed in this article are solely those of the authors and do not necessarily represent those of their affiliated organizations, or those of the publisher, the editors and the reviewers. Any product that may be evaluated in this article, or claim that may be made by its manufacturer, is not guaranteed or endorsed by the publisher.

## Footnotes

^1^ http://dbm.neuro.uni-jena.de/cat/

^2^ http://www.fil.ion.ucl.ac.uk/spm/software/spm12/
